# Cohort study of prevalence and phenomenology of tremor in dementia with Lewy bodies

**DOI:** 10.1007/s00415-013-6853-y

**Published:** 2013-02-12

**Authors:** Marco Onofrj, Sara Varanese, Laura Bonanni, John-Paul Taylor, Angelo Antonini, Enza Maria Valente, Simona Petrucci, Fabrizio Stocchi, Astrid Thomas, Bernardo Perfetti

**Affiliations:** 1 Department of Neuroscience and Imaging, University G. d’Annunzio of Chieti-Pescara, Via dei Vestini, 66100 Chieti, Italy; 2 Aging Research Center, Ce.S.I. “Gabriele d’Annunzio” University Foundation, Chieti, Pescara Italy; 3 Department of Neurology, Division of Movement Disorders, New York University, NY, USA; 4 Newcastle University (Institute for Ageing and Health), Newcastle upon Tyne, UK; 5 IRCCS San Camillo, Venezia Lido, Italy; 6 Casa Sollievo Della Sofferenza Hospital, Mendel Institute, Rome, Italy; 7 Institute for Research and Medical Care, San Raffaele, Rome, Italy; 8 Department of Physiology and Pharmacology, Sophie Davis Medical School, City College of New York, NY, USA

**Keywords:** Dementia with Lewy bodies, Parkinson’s disease, Tremor, EMG

## Abstract

To study prevalence, specific patterns and response to treatment of tremor in dementia with Lewy bodies (DLB), in comparison with other tremulous disorders prevalence, qualitative and quantitative features of tremor were studied in an incident cohort of 67 dopaminergic treatment naive DLB, 111 Parkinson’s Disease (PD) and 34 Essential Tremor (ET) patients. Tremulous DLB patients (tDLB) were compared with tremulous PD (tPD) and ET patients and followed for 2 years. Double blind placebo-controlled acute drug challenge with l-Dopa and alcohol was performed in all ET, 24 tDLB and 27 tPD. Effects of dopaminergic chronic treatment in all tDLB and tPD patients and primidone in 8 tDLB were also assessed. Tremor occurred in 44.76 % of DLB patients. The tDLB patients presented a complex pattern of mixed tremors, characterized by rest and postural/action tremor, including walking tremor and standing overflow in 50 % tDLB. Standing tremor with overflow was characteristic of tDLB (*p* < 0.001). Head tremor was more frequent in tDLB than tPD and ET (*p* = 0.001). The tDLB tremors were reduced by acute and chronic dopaminergic treatments (*p* < 0.01) but not by alcohol or primidone. Tremor occurs commonly in DLB patients with a complex mixed tremor pattern which shows a significant response to acute and chronic dopaminergic treatments. Recognizing that there is a clinical category of tremulous DLB may help the differential diagnosis of tremors.

**Electronic supplementary material:**

The online version of this article (doi:10.1007/s00415-013-6853-y) contains supplementary material, which is available to authorized users.

## Introduction

The last consensus on diagnostic criteria for dementia with Lewy Bodies (DLB), states that “tremor is less frequent than in Parkinson’s disease (PD)” [1] but does not detail the types and relative prevalence of tremor, despite earlier studies reporting a prevalence of 40 % for rest and 30 % for action (kinetic-postural) tremor [2, 3].

Generally rest tremor is considered specific for PD, while postural and action (or intentional) tremor are attributed to essential tremor (ET), although exceptions to this rule are clearly reported [4–6]. The prevalence of these tremors have not been investigated in DLB after the early reports, nor was described the prevalence of other types of tremor which are considered infrequent but specific to PD, as head and face tremors [7, 8], re–emergent [6, 9], pseudo-orthostatic or standing tremor [5, 10–13]. In addition, while there are some small studies reporting an improvement of general motor features in DLB with acute l-Dopa challenge [14], the specific response of tremors to treatments has been investigated in only one early study [15].

The clarification of the different tremor types and response to treatment in DLB could improve clinical recognition of DLB, but mostly, understanding tremor in DLB, would provide clarity to recent controversial debates [16–24] on the long term outcome of patients putatively affected by ET. Several reports showed that some ET patients, during follow-up may present with additional PD features, dopamine transporter capitation abnormalities, or cognitive abnormalities, and may present with Lewy bodies at the autopsy [16–21]. These observations were, more or less dismissively, challenged in three different editorials [22–24]. Yet, these discussions did not consider at all that the action (kinetic, postural) tremors, of DLB may be erroneously attributed to ET.

Therefore, our study aims to show characteristics and treatment sensitivity of tremor in DLB in comparison with tremor in PD and ET, in order to be of help to neurologists attempting categorization of patients.

An incident cohort of DLB patients was evaluated and followed for two years and compared with two cohorts of incident PD and ET patients. Detailed and extensive evaluation of any tremors present was carried out and response to acute and chronic treatments was also investigated.

## Methods

All new referrals to our movement disorders and memory clinics in the years 2005–2009, (202 patients) diagnosed with DLB, PD, or ET, according to the accepted clinical criteria [1, 4, 25] were enrolled in the study. Exclusion criteria were any prior exposure to neuroleptic drugs, the presence of dystonia and the presence of classic orthostatic tremor. The PD patients with dementia (PDD) were excluded because of the confounding effects of treatments.

All patients tested negative for the G20195 mutations in the LRRK2 gene [26] as a high prevalence of mixed tremor has been described in this condition [27]. All patients were followed for a minimum of two years to confirm/challenge diagnosis.

The study was approved by our local ethical committee and was carried out according to the declaration of Helsinki and subsequent revisions [28]. Patients (or caregivers) signed a written informed consent and authorization was obtained for disclosure (consent-to-disclose) of any recognizable persons in videos. All patients received a full neurological, neuropsychological, neuroimaging and neuropsychiatric evaluation, as reported in Online Resource 1, and as described in previous studies on the same cohort [29–32]. Observation and recording of tremors were performed only in dopaminergic drugs naive patients (all DLB and 52 PD patients) or after withdrawal of l-Dopa (48 h) or dopamine agonist (72 h) treatments (13 PD patients).

Tremor was rated using the Tremor Research Group Rating Scale (TRGRS) [6] quantified clinically according to the TRGRS items. Face and jaw tremor were clustered in a single item, tongue tremor was omitted as this was not observed, and items 10 and 11 (hand writing and holding pencil) were omitted as these items were not collected in all patients. Two supplementary categories, absent in the TRGRS rating scale were also applied: re-emergent tremor of the arm indicating “delayed re-emergence of rest tremor when the arm was postured” [9] and standing overflow indicating overflow of rest or postural tremor, when standing, to body districts not primarily affected by the tremor. Standing tremor was defined according to the TRGRS scale, and indicated the pseudo-orthostatic tremor with the low frequency of parkinsonian tremor or ET, described in PD or genetic PD [10–13]. Tremors frequencies and amplitudes were also quantified by Electromyography (EMG) as described in our previous papers [11, 12, 33] and in Online Resource 1.

Acute drug challenge was performed in 34 ET, 24 DLB and 27 PD patients. Acute treatments were evaluated in the three groups with blinded procedures. Alcohol test and acute l-Dopa challenges were performed according to the described protocols [15, 34–36]. An alcohol test was performed in all ET, PD and DLB patients with tremor, in blinded protocols. A target of 0.8 ‰ (0.8 g/L) alcohol was reached by administering in 5 min, 8 fluid oz of orange juice with artificial nonalcohol Rum flavour (placebo) or orange juice with 40 % alcohol Rum. The needed amount of alcohol was calculated for each patient according to published body water formulas. The TRGRS rating was performed by examiners unaware of the administered substance 30–45 min after the administration according to published observations on maximal tremor responses to ethanol administration [34].

Double blind cross-over acute l-Dopa challenges (single oral adminiostration l-Dopa/carbidopa 250/25 mg) [36] were performed in patients never exposed to l-Dopa with the TRGRS rated by examiners 90–110 min after drug or placebo intake.

Primidone effect (250–500 mg/day) was evaluated in eight DLB patients, who were taking this drug for presumed ET prior to referral to our clinics. In these patients TRGRS ratings and tremor assessments were performed during primidone treatment and two weeks after withdrawal.

During the follow-up period all DLB and PD patients received oral l-Dopa treatment and other treatments according to their needs. Tremor ratings were repeated every two to four months; the chronic treatment effect evaluation was performed six months after the initial assessment.

### Statistics

Demographic, clinical and neuropsychological/neuropsychiatric differences between groups and time of evaluation were estimated using either analysis of variance for continuous variables (One-way ANOVA; ANOVA for repeated measure) or non-parametric procedures as appropriate (Wilcoxon test). Between-groups differences in the prevalence of the specific types of tremor were investigated using the chi-square test. Stepwise logistic regression and ROC analyses were used to assess the best group predictor among the types of tremor. Post hoc clustering of TRGRS scores was applied to treatment effect evaluations in order to simplify graphic rendering of data.

Correlations between tremor scores and neuropsychological tests were not applied because the range of scores for cognitive variables clustered specifically in the DLB population. All analyses were conducted using statistical package SSPS 16.

## Results

### Patients characterization

Among the 202 new referrals to our movement disorders and memory clinics during the years 2005–2009, 111 were initially diagnosed with PD, 61 with DLB and 40 with ET.

**Fig. 1:**
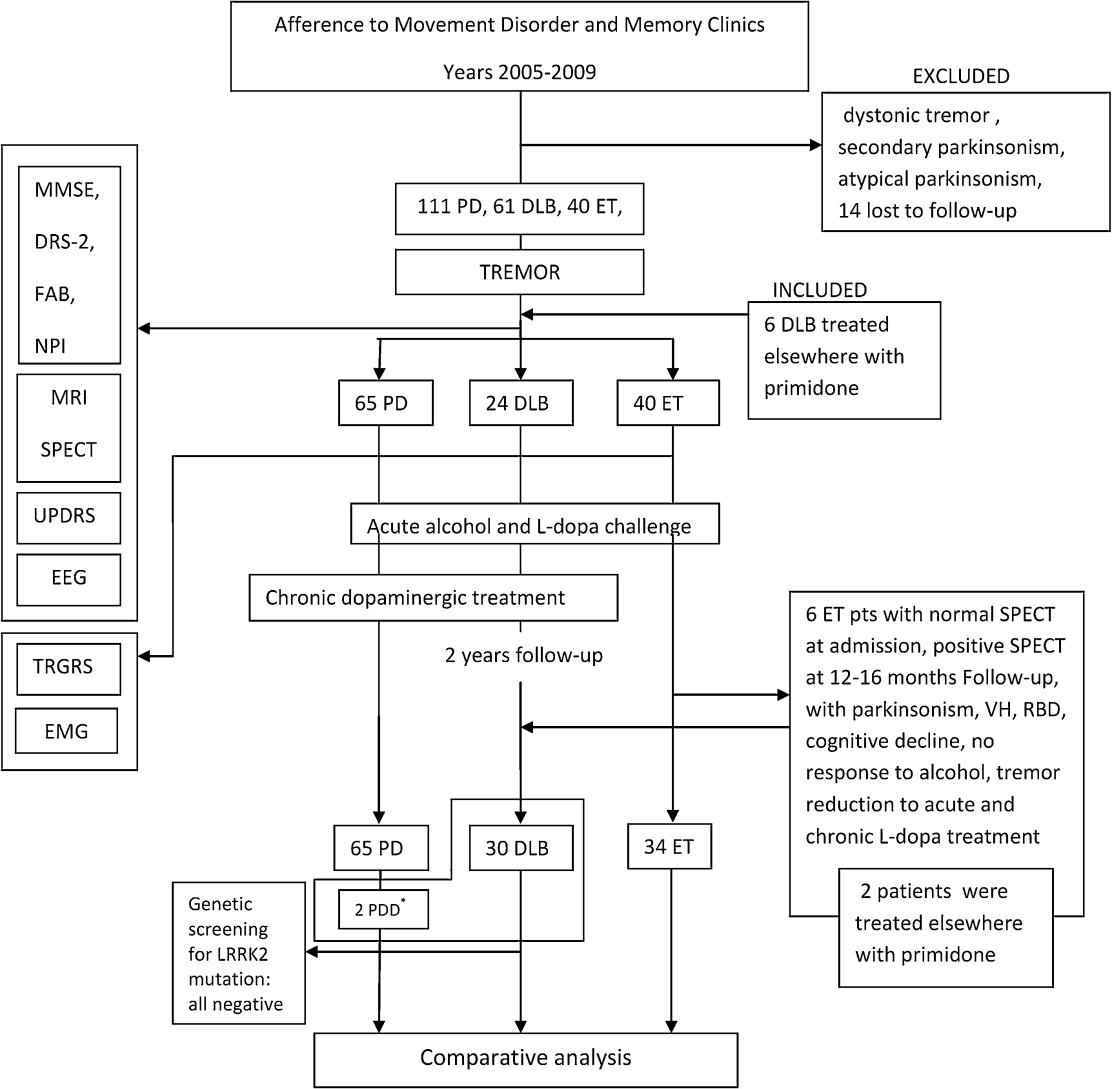
Flow chart of the study: referral to clinics, selection of patients according to instrumental evaluation proposed by Consortium Consensus criteria, and drug effect assessments. The comparative analysis was performed only in patients whose diagnosis was not challenged during the follow-up. The *asterisk* indicates that the two patients who developed PDD during follow-up were considered in the 65 patients of the PD group

Within two years from the ET diagnosis, six patients were re-classified as DLB due to the appearance of cognitive decline with all core and supportive consensus elements [1], including REM sleep behaviour disorder (RBD), hallucinations, EEG abnormalities, and cognitive fluctuations. In these patients DLB categorization, instead of PD with Dementia (PDD), was considered acceptable despite the fact that tremor had appeared before or was concomitant with cognitive decline, as the tremor observed at first referral was purely intentional-kinetic and all six had been addressed to referral as ET patients and two of them were temporarily treated with primidone until our re-evaluation. Six more patients were treated with primidone according to putative ET diagnosis, but in these patients the diagnosis of DLB was completed at first referral. Figure 1 shows the study design-flow chart with categorization of patients.

Thus, a total of 67 patients were ultimately classified as DLB and 34 as ET. Two PD patients, presenting with rest/action and standing tremor, developed cognitive impairment and neuropsychiatric symptoms and were reclassified PDD [37] .

Demographic and clinical characteristics, with statistical comparisons, of the three groups at baseline and follow up are reported in Table 1. Age, results of cognitive tests and the degree of cognitive decline at follow-up significantly separated DLB patients from PD and ET. Onset of tremor, onset of dementia and other clinical characteristics in the eight patients treated with primidone prior to our re-classification into DLB was not statistically different in comparison to the other 22 tremulous DLB patients.

**Table 1:** Demographic and clinical characteristics of groups

	DLB (*n* = 67)	PD (*n* = 111)	ET (*n* = 34)	*p* (between)
Age	72.15 (5.43)	66.52 (8.84)	67.79 (3.73)	<0.005^a^
Gender (F/M)	35/31	51/60	16/18	NS
Disease duration	2.86 (0.95)	4.07 (3.1)	4.7 (1.91)	<0.001^a^
UPDRS III				
Baseline	26.98 (5.14)	24.47 (12.4)	NA	<0.001^b^
Followup	21.61 (5.28)	18.01 (11.44)	NA	
*p* (within)	<0.001	<0.001		
MMSE				
Baseline	18.11 (3.27)	28.07 (1.5)	28.7 (1.5)	<0.001^a^
Followup	12.95 (2.78)	26.82 (1.93)	27.91 (2.1)	
*p* (within)	<0.001	<0.001	NS	
DRS2				
Baseline	84.05 (12.82)	136.89 (7.08)	136.29 (2.6)	<0.001^a^
Followup	61.61 (12.85)	132.04 (8.73)	135.78 (3.2)	
*p* (within)	<0.001	<0.001	NS	
FAB				
Baseline	12.41 (1.53)	17.09 (1.13)	17.54 (0.8)	<0.001^a^
Followup	9.67 (1.36)	15.48 (1.3)	17.46 (1.1)	
*p* (within)	<0.001	<0.001	NS	
CAF				
Baseline	2.95 (1.4)	0.05 (0.42)	0	<0.001^a^
Followup	5.13 (1.31)	0.24 (1.03)	0	
*p* (within)	<0.001	<0.001	NA	
NPI				
Baseline	20.74 (1.6)	7.6 (2.84)	1.8 (1.7)	<0.001^b,d^
Followup	28.74 (1.60)	8.61 (4.14)	1.9 (1.5)	
*p* (within)	<0.001	<0.001	NS	
RBD (n/%)				
Baseline	58 (86.6)	47 (42.3)	0	<0.001^c^
Followup	62 (92.5)	70 (63.1)	0	
*p* (within)	NS	0.033	NA	
VH(*n*/%)				
Baseline	49 (73.1)	0	0	NA
Followup	62 (92.5)	31 (27.9)	0	
*p* (within)	NS	NA	NA	

All values represent mean (SD, when not otherwise stated). Reported *p* values compare between groups (DLB, PD and ET) and within groups (baseline and follow-up). The* p* values have been calculated using Oneway ANOVA with Bonferroni corrections for parametric variables and *χ* ^2^ for categorical variables. Disease duration indicates evidence of cognitive decline in DLB

*MMSE* Mini Mental State Examination, *NP * neuropsychiatry inventory, *FAB* frontal assessment battery, *DRS-2* Dementia Rating Scale-2, *CAF* clinician assessment of fluctuation, *UPDRS-III* Unified Parkinson’s Disease Rating Scale-subscale III, *H/Y* Hoehn/Yahr staging, *RBD* REM sleep behavior disorders, *VH* visual hallucinations (also including benign VH), *NA* not applicable, *NS* not significant

^a^DLB different from PD and ET

^b^ET different from PD and DLB

^c^DLB different from PD

^d^PD different from ET

### Prevalence of tremor

Tremor was observed in 30 DLB and 65 PD patients. Overall, patients with tremor represented 44.77 % of the DLB cohort (tDLB) and 58.56 % of the PD cohort (tPD). Their characteristics at baseline and follow up are reported in Table 2; there were minor differences between tDLB and nontremulous DLB (age, disease duration and cognitive function) but no differences between PD subgroups.

**Table 2:** Comparison between tremulous and non-tremulous patients within the DLB and PD groups

	DLB (*n* = 67)			PD (*n* = 111)		
	Tremor (30)	Non-tremor (37)	*p*	Tremor (65)	Non-tremor (46)	*p*
Age	73.9 (5.17)	70.74 (5.3)	0.017	64.63 (9.82)	69.19 (6.44)	0.007
Disease duration	2.13 (0.86)	3.45 (0.5)	<0.001	4.3 (3.31)	5.23 (2.86)	NS
UPDRS III	25.5 (4.61)	28.18 (5.3)	0.032	23.52 (13.04)	25.82 (11.43)	NS
MMSE	19.03 (2.56)	17.37 (3.61)	0.039	28.09 (1.72)	28.04 (1.31)	NS
DRS2	87.96 (7.69)	80.89 (15.19)	0.024	136.16 (8.25)	137.91 (4.9)	NS
FAB	12.36 (1.69)	12.45 (1.42)	NS	17.03 (1.22)	17.17 (0.99)	NS
CAF	3.2 (1.34)	2.75 (1.44)	NS	0.09 (0.55)	0	NS
NPI	20.7 (1.55)	20.78 (1.66)	NS	7.27 (3.14)	8.19 (2.26)	NS
RBD (*n*)	28	30	NS	29	18	NS
VH (*n*)	24	25	NS	0	0	NA

All values represent mean (SD). *p* values have been calculated using Oneway ANOVA with Bonferroni corrections for parametric variables and *χ* ^2^ for categorical variables

*MMSE* Mini Mental State Examination, *NP* neuropsychiatry inventory, *FAB* frontal assessment battery, *DRS-2* Dementia Rating Scale-2, *CAF* clinician assessment of fluctuation, *UPDRS-III* Unified Parkinson’s Disease Rating Scale-subscale III, *H/Y* Hoehn/Yahr staging, *RBD* REM sleep behavior disorders, *VH* visual hallucinations (also including benign VH), *NA* not applicable, *NS* not significant

### Phenomenology of tremors and relative prevalence

Table 3 shows severity and prevalence of tremor types in the tDLB, tPD and ET groups based on the TRGRS scores. The table shows that the full range of tremors, including head and face tremors, was observed with different severity and prevalence in the three groups of patients. Limb tremor was more frequently bilateral in tDLB as compared to tPD (73.3 % vs. 9.2 %, *χ* ^2^ = 40.6; *p* < 0.001).

**Table 3:** Severity of tremor types as per TRGRS scores

TRGRS	DLB (*n* *=* 30)				tPD (*n* = 65)				ET (*n* *=* 34)			
	% (*n*)	Mean ± SD	Freq-Hz	Side B % (*n*)	% (*n*)	Mean ± SD	Freq-Hz	Side B % (*n*)	% (*n*)	Mean ± SD	Freq-Hz	Side B % (*n*)
1. Head	27 (8)	1.0	2.5–4.8	–	3 (2)	1.0	2.4–4.9	–	29 (10)	1.30 ± 0.5	2.5–5.2	–
2. Face	43 (13)	1.31 ± 0.5	4.1–7.2	–	25 (16)	1.31 ± 0.5	4.2–6.9	–	–	–	–	–
3. Voice	–	–	–	–	–	–	–	–	17 (6)	1.25 ± 0.5	NA	–
4. a. Rest arm	87 (26)	1.77 ± 0.6	4.9–7.2	57 (16)	82 (53)	2.08 ± 0.8	4.9–7.1	8 (4)	9 (3)	1.0	5.2–7.4	– (3)
b. Forward arm	57 (17)	2.24 ± 0.4	4.3–7.5	59 (10)	46 (30)	1.50 ± 0.7	4.6–7.4	10 (3)	100 (34)	1.94 ± 0.7	5.4–7.4	100 (34)
c. Lateral arm	57 (17)	2.00 ± 0.8	4.8–7.2	59 (10)	15 (10)	1.50 ± 0.7	4.3–7.3	10 (1)	100 (34)	2.00 ± 0.8	5.2–7.3	100 (34)
b-c* reemergent	57 (17)				19 (12)							
d. Kinetic	60 (18)	1.89 ± 0.7	4.9–7.3	89 (16)	15 (10)	1.60 ± 0.7	4.7–7.1	10 (1)	100 (34)	1.85 ± 0.6	5.1–7.1	100 (34)
e. Walking	80 (24)	2.25 ± 0.8	5.1–7.5	68 (17)	72 (47)	1.74 ± 0.7	4.3–7.6	26 (12)	–	–	–	–
5. Trunk	27 (8)	1.37 ± 0.5	4.0–7.2	–	5 (3)	1.33	4.1–7.0	–	9 (3)	1.66	5.1–7.0	–
6. Action leg	53 (16)	1.12	4.2–6.9	44 (7)	3 (2)	1.0	4.3–7.4	0 (0)	56 (19)	1.37 ± 0.5	5.3–7.3	100 (19)
7. Rest leg	53 (16)	1.31 ± 0.5	4.2–7.0	75 (12)	45 (29)	1.31 ± 0.5	4.1–7.2	31 (9)	3 (1)	1.0	5.2–7.3	– (1)
8. Standing	53 (16)	2.25 ± 0.7	4.6–7.3	–	5 (3)	2.33	5.1–7.2	–	3 (1)	1.0	5.1–7.1	– (1)
*with overflow	47 (14)				3 (2)							
9. Spiral drawing	50 (15)	1.87 ± 0.6	NA	67 (10)	15 (10)	1.40 ± 0.5	NA	20 (2)	100 (34)	2.18 ± 0.8	NA	100 (34)
10. Water pouring	47 (14)	1.71 ± 0.7	NA	71 (10)	12 (8)	1.25 ± 0.5	NA	25 (2)	100 (34)	2.12 ± 0.8	NA	100 (34)

Item of the TRGRS are reported on the left, face tremor in the table is the average for each patient of face and jaw tremor. Tongue tremor was not observed. Items 10 and 11 are omitted as not rated in all patients

Percentages represent the prevalence of each item in the group (score > 1) and are approximated to the following unit if >0.4

The re-emergent item 4 b-c* indicates forward and lateral arm tremor appearing not earlier than 5 s from acquiring the posture. Standing *with overflow indicates spreading of tremor from affected arms to other body parts when standing. These supplementary items were not specifically rated as not applicable, but amplitude scores were reported in items 4* and 8

TRGRS scores are reported as mean ± SD (standard deviation). SD scores are not reported if *n* < 4.  Side is coded as bilateral B versus unilateral predominant (TRGRS score one side being >50 % of the score of the controlateral side)

*NA* not applicable available

**Fig. 2:**
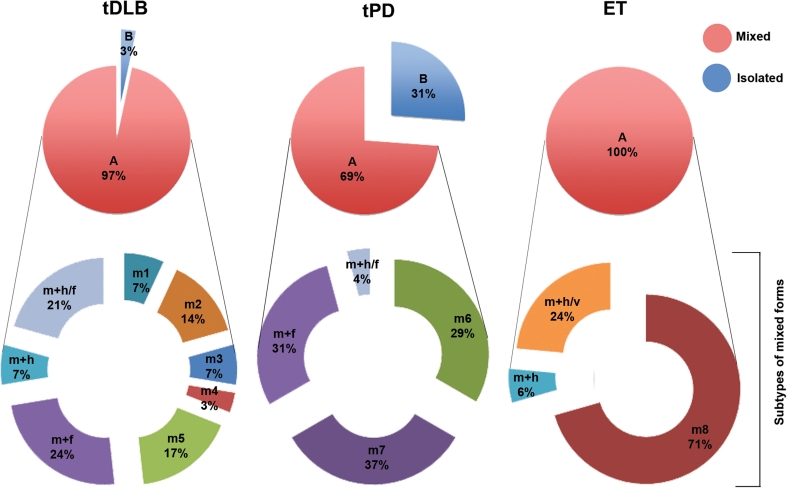
Graphic representation of prevalence of mixed tremor and overlap of tremors in tDLB, tPD and ET. Notice the complexity of mixed forms of tremor in tDLB as compared to tPD and ET. The *circle* or “*pie*” graph on the *upper* half shows the general categories of mixed (any combination) **a** or isolated forms of tremor, (rest) **b**. The ring or exploded “doughnut” graph below each *pie* represents separate subcategories of tremors in the same populations, as marked by following symbols: *m* mixed, **m1**: sum of action postural rest arm, action leg, + walking tremor; **m2**: rest + action + walking tremor; **m3**: rest + action + postural + walking tremor; **m4**: rest + action + walking + standing tremor; **m5**: rest + action + walking + postural arm + standing tremor; **m6**: rest + walking tremor; **m7**: rest + walking + postural tremor; **m8**: action + postural tremor; **m** **+** **f**: mixed + face tremor; **m** **+** **h**: mixed + head tremor; **m** **+** **h/f**: mixed + head + face tremor; **m** **+** **h/V**: mixed +head + voice

The ET tremor was bilateral as a result of selection criteria [3,4]. The overlap of tremor types in the same patients is graphically represented in Fig. 2, and Table 3 shows that tDLB was characterized by several tremor types including overlapping mixtures of head and face, rest, postural, action (spiral drawing, pouring water) and walking (re-emergence of rest arm tremor when walking) tremor. Almost all of the tDLB patients (97 %) expressed more than one type of tremor.

Head and face tremor was more frequent in tDLB compared to tPD (26.7 % vs. 3.1 % for head tremor *χ* ^2^ = 12.1 *p* = 0.001; 40.3 % vs. 24.6 % for face tremor; *χ* ^2^ = 3.4 *p* = 0.06). Voice tremor (bleating voice) was observed only in 6 ET (17.6 %).

For tDLB, 44.9% presented with a complex pattern of mixed tremor which consisted of rest, postural, and action tremor and these were associated with walking and/or standing tremor.

This mixed tremor appeared as hand pill-rolling tremor at rest and when walking but action tremor was also evident when patients attempted to perform the spiral drawing/pouring water TRGRS tasks or when the patient was asked to write.

Standing posture elicited tremor of lower limbs (standing or pseudo-orthostatic tremor) in 53.3 % of tDLB patients, and 4.6 % of PD patients (*χ* ^2^ = 24.4; *p* < 0.001). Leg tremor disappeared when walking whilst tremor of arms appeared. Only one ET patient showed standing tremor, but no walking tremor. Two PD patients with standing tremor presented during follow-up with features of PDD.

A specific characteristics of the mixed tremor observed in DLB patients was an overflow phenomenon elicited by the standing posture with outstretched arms, characterised by the diffusion of tremor to all the different body parts; this overflow was observed in 46.6 % of DLB patients but only in 3.1 % of PD patients (*χ* ^2^ = 27.8; *p* < 0.001). This overflow tremor appeared as hand pill-rolling tremor at rest and when walking but kinetic tremor was also evident when patients were attempting the spiral drawing/pouring water TRGRS tasks or when the patient was asked to write. When these patients were evaluated in a standing position with outstretched arms, tremor appeared in the hands with a latency of 3–15 s and a progressive overflow to lower limbs was observed in 5–30 s. The latency of rest tremor reappearance when acquiring the outstretched arm position was 10.2 ± 4.63 s which contrasted with the almost immediate occurrence of postural tremor in ET, at 1.68 ± 2.27 s (Wilcoxon tests, *Z* = −2.93, *p* = 0.003).

**Fig. 3:**
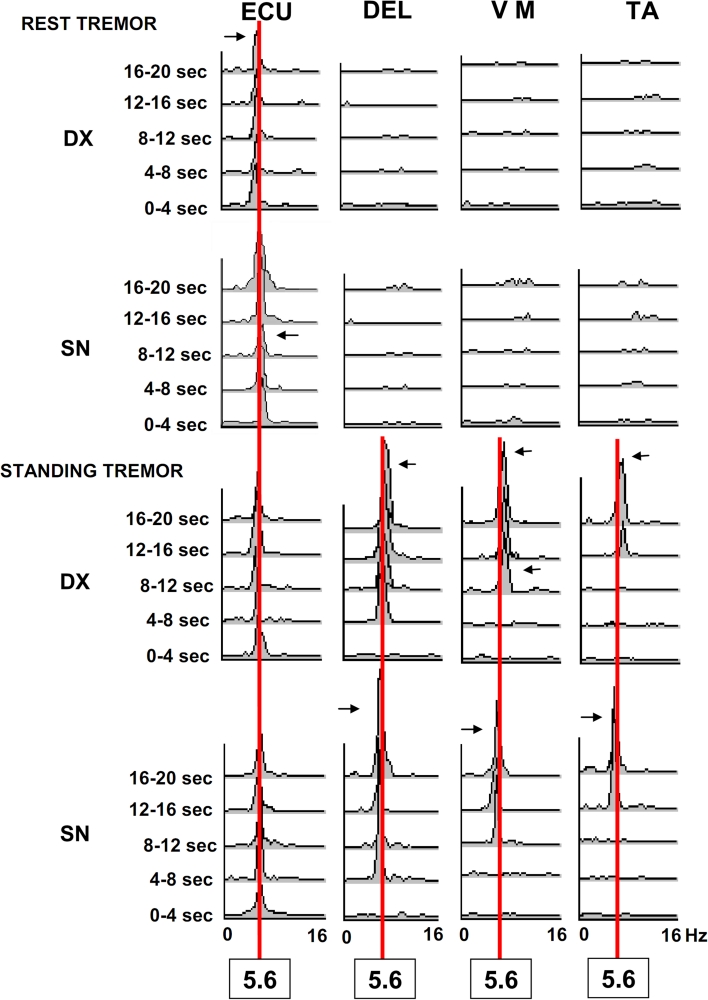
Frequency analysis and power spectra of tremor at rest and during standing with wing beating arm posture in a patient affected by DLB. EXTENSOR CARPI ULNARIS = ECU; DELTOID = DEL; VASTUS MEDIALIS = VM; TIBIALIS ANTERIOR = TA Horizontal scales represent frequencies in Hz, (0–16 Hz) amplitude of random noise frequencies are 0.2–0.6 μV^2^. A vertical red line connects 5.6 Hz frequencies, which is the specific tremor frequency in this patient. Horizontal arrows point to minor frequency variations (range 0.5–01 Hz) of the peak amplitude. Notice overflow during standing posture for the same districts involved by the rest tremor to other body districts

Figure 3 shows the EMG pattern of progressive overflow. The TRGRS scores or tremor frequencies were not statistically different when rest, postural or action tremors were compared with tremor appearing in standing position (*F* = 0.57; *p* = 0.68 for TRGRS score; *F* = 0.46; *p* = 0.76 for frequencies).

Combinations of different types of tremor were observed in 45 PD patients but 29 % of these patients presented simply with a combination of rest and walking tremors. (Fig. 2).

The classic walking tremor was observed in 80 % of tDLB and 72.3 % of tPD patients but in none of ET patients.

Isolated arm rest tremor was observed in only 3.3 % of DLB patients compared with 30.8 % of PD (*χ* ^2^ = 5.6; *p* = 0.02). The tDLB patients did not present with isolated postural or action tremor nor the combination of the two; instead this tremor pattern was observed in 70.6 % of ET patients. Tremor frequencies did not differentiate specific tremor patterns although the head tremor had a significantly lower frequency than rest and postural (Wilcoxon test, Z = −3.6, *Z* = −3.1, respectively; *p* < 0.001; Table 3). Additional information is detailed in Online resource 2.

### Regression analysis

In a stepwise regression analysis of the types of tremor observed, the standing tremor proved to be the best predictor of membership in a disease group (*B* = −1.80; Wald = 17.88; *p* < 0.001; 95 % CI: 0.071, 0.38). Specifically, the presence of standing tremor correctly predicted 46.7 % of tDLB patients, and this percentage increased to 53.5 % when standing and rest tremors entered together in the model (for rest tremor, *B* = −0.47; Wald = 4.74; *p* = 0.03; 95 % CI: 1.05, 2.47). Conversely, the absence of standing tremor itself correctly predicted 96.9 % of tPD suggesting that standing tremor is rare in PD. Roc analysis is reported in Online resource 3.

### Effect of treatments

#### Acute treatments

**Fig. 4:**
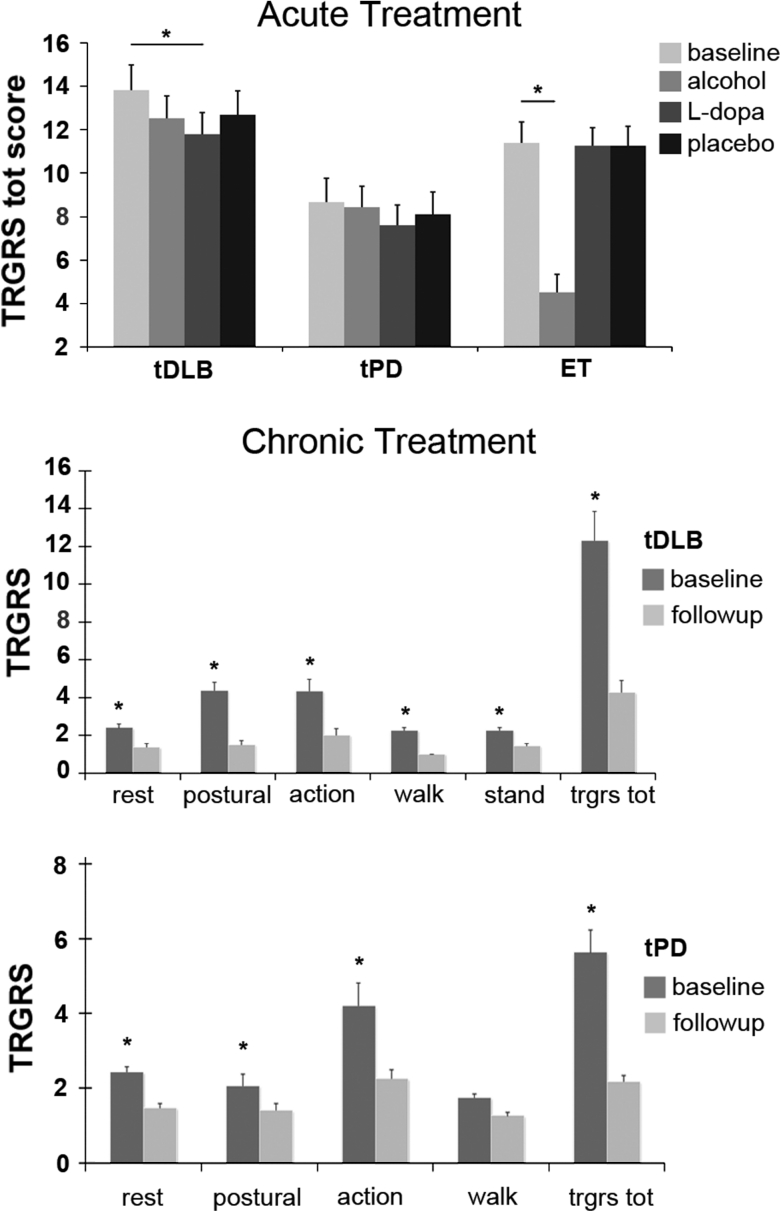
Response to acute drug challenge and chronic treatment. The scores are expressed as mean TRGRS scores. *Bars* represent the SE. * *p* < 0.01 **a**: acute treatment. Total TRGRS scores. Improvement after l-Dopa administration was significant only in tDLB group (*p* < 0.001). Acute alcohol test improved tremor only in ET (*p* < 0.001). **b** Tremor in chronically treated patients clustered scores. Rest tremor is the sum of head, face, rest arms and rest leg items; postural tremor is the sum of forward arm and lateral arm items; action is the sum of kinetic, action leg, spiral and water items, mixed tremor defines any combination of rest, postural, action, standing and walking tremors

l-Dopa significantly reduced tremor only in the tDLB group (effect of drug, *F* = 8.56, *p* = 0.001; mean difference on TRGRS total score = 2.05, SE = 0.41, 95 % CI 0.84–3.24, *p* < 0.001 Bonferroni corrected) while it was ineffective in ET patients. It did improve tremor in a proportion of tPD patients but this improvement was not significant. An acute alcohol test significantly reduced tremor in ET (effect of drug, *F* = 104.7, *p* < 0.001; mean difference on TRGRS total score = 6.88, SE = 0.37, 95 % CI 5.82–7.94, *p* < 0.001 Bonferroni corrected) but was ineffective in tDLB and tPD patients. Acute drug response is depicted in Fig. 4a as a change in total TRGRS score, as no differences in body distribution or the type of tremors were noted following drug administrations.

#### Chronic treatment

Chronic dopaminergic treatments administration improved all the types of tremors in tDLB (Wilcoxon test, *Z* values range −2.9 and −4.2; *p* < 0.005) and all but action tremor in tPD (Wilcoxon test, *Z* values range −2.9 and −6.4; *p* < 0.005). The typical standing tremor observed in tDLB improved from a mean TRGRS score of 2.25 to a mean TRGRS score of 1.42 after chronic l-Dopa administration. Total disappearance of tremor was observed in 12 tDLB and 14 tPD patients.

Effect of chronic dopaminergic treatment on clustered TRGRS items is illustrated in Fig. 4b. Mixed tremor with overflow on a standing position disappeared in one PD and in 12 out of 15 DLB patients. In all DLB patients TRGRS scores for rest, postural or intentional tremor components during l-Dopa treatment were reduced by 1–2 points on the TRGRS. Comparison of scores prior to treatment and scores after six months of treatment demonstrated that there was a 1–2 point reduction in the TRGRS score, corresponding to reduction of 50–90 % for rest, 60–90 % for postural and 60 % for the intentional component. Head and chin tremor was reduced from a score of 2–3 to 1 point in 4 DLB and 1 PD patient, and was abolished (from score 2–0) in 1 DLB and two PD patients, while these tremors were not modified in three DLB and six PD patients.

During chronic l-Dopa treatment (mean daily dose 300 ± 45.5 mg), two patients (1 DLB, 1 PDD), whose mixed tremor had been suppressed, showed a residual small amplitude cortical mini-polymyoclonus in the fingers, evident in outstretched horizontal position of the arms, already described in association with Lewy body pathology [3] which was undetectable before l-Dopa administration, due to superimposition of postural tremor components.

#### Primidone

The eight DLB patients who received primidone (125 b.i.d. in 6, 250 mg b.i.d. in two) prior to referral to our clinics presented with mixed tremors, including postural action tremor with TRGRS score from 2 to 4 and variable rest tremor (TRGRS score 1–3). Four of them presented with standing tremor (TRGRS score 2,3). Primidone withdrawal was not followed by tremor worsening (Wilcoxon test, *Z* = −1.13; *p* = 0.25) with no differences between tremor types. In the same patients chronic l-Dopa therapy significantly reduced tremor scores (mean Total TRGRS score difference = 14.1; Wilcoxon test, *Z* value = −2.52, *p* = 0.012). Online Resource 4 shows detailed results.

## Discussion

Our cohort study showed that tremor occurred in 45 % of DLB patients compared to 59 % of PD patients; thus it confirmed that tremor is less frequent in DLB than PD but challenged the assumption that tremor is rare in DLB.

Thus, our findings suggest that DLB, indeed, consists of tremulous and non tremulous variants.

Furthermore our data shows that tremor in DLB patients is in high prevalence a bilateral mixed tremor (postural, action, standing with head tremor) and we demonstrated that it shows responsiveness to l-Dopa treatment, but do not respond to primidone or alcohol.

### Strengths and limitations

The comprehensive nature of our assessments, including the application of established diagnostic criteria [1] and of SPECT and EEG assessments, of treatment protocols with l-Dopa and alcohol, and of a wide spectrum of neuropsychological test batteries and the two year follow-up ensured a high degree of diagnostic certainty, within the limit of clinical studies not supported by neuropathology [3, 15, 29, 30]. In addition we could exclude that G20195 mutations in the LRRK2 gene, which associate with tremors [27], were present in our cohort population.

A definite limitation is that our findings could not be supported by neuropathological ascertainments, as no ascertainment exists for ET.

However, the majority of clinical studies, same as ours, could not benefit from the support of neuropathology, but yet were considered staminal studies [3, 14, 18].

A second possible limitation is the ascertainment bias of patients referred to our centre, as we included all patients who were referred to both our movement disorders and memory clinics following procedures that underline our specific scientific interest [29–32]. However the prevalence of tremor in our cohort (45 %) was not different from prevalence rates found in earlier studies [2, 3] conducted in memory clinics.

### Characterisation of tremulous DLB (tDLB)

The tDLB patients had shorter disease duration and better MMSE and DRS2 scores than non-tremulous ones and this may be due to the fact that individuals with the obvious symptom of tremor are referred earlier in their disease course. The tDLB patients were also modestly older than the nontremulous ones. We believe, however, that older age does not indicate that senile tremor was overlapping to DLB features, as the phenomenology and opposite responses to l-Dopa and alcohol evidence that tremor in tDLB was characterized by specific patterns.

In detail beyond rest tremor, the different tremor patterns observed in tDLB were head and face [7, 8], postural and action, re-emergent [9], walking and standing (also called pseudo orthostatic) [5, 11, 12] tremors, which are reported to occur in PD less frequently than rest tremor. In addition, the standing/pseudo-orthostatic tremor [5, 11–13] appeared to be particularly common in tDLB patients. The standing posture revealed one further feature of this mixed tremor: there was overflow from arms to other body districts in a few seconds which might be considered a particularly severe example of the specific re-emergent postural tremor described in PD [8], which is different from postural tremor of ET, because in ET, posture elicits tremor immediately, while the re-emergent tremor appears with a delay when acquiring the posture (10 ± 4 in our cohort).

Due to the complex and variable expression of tremors in DLB the regression analysis was weak at identifying a single tremor type which was predictive of DLB although the presence of standing tremor correctly predicted (regression and ROC analysis) in about half of the tDLB patients, with the same tremor being absent in ET and very rare in PD.

Differential diagnosis was helped by identification of 4 tremor types: walking, re-emergent and standing tremor were observed only in tDLB and tPD patients while the voice tremor was only observed in ET patients.

### Treatment of tremors in DLB

The second relevant finding of our cohort study was that in DLB all types of tremors including head, mixed and standing tremor showed a significant response to acute l-Dopa challenge or chronic l-Dopa treatment and were not modified by alcohol administration according to standard tremor assessment protocols [35, 36], whereas ET was reduced by alcohol. This not only has implications for clinical practice but also for the aetiology of tremor in DLB as it suggests that tremor in DLB, when present, arises from dysfunction of the dopaminergic system and is a unitary disorder specific to this condition rather than a variant of ET or a senile tremor, or is due to concomitant occurrence of both ET and DLB.

Further support to this conclusion was obtained in the present study by the observation of the effect of primidone withdrawal in eight patients who had received primidone because of the initial diagnosis of ET but who presented with all core and supportive consensus elements of DLB. Primidone withdrawal had no effect on tremor in these patients, also alcohol administration had no effect, while their tremor was significantly reduced by l-Dopa (Online Resource 4).

This minor part of our study, restricted to a small number of patients, together with the other data, suggests a further clinically relevant conclusion: the mixed pattern of tremor (given the prominent action-postural components) in DLB could be the source of possible misdiagnosis.

Some DLB patients were inappropriately, yet temporarily, classified as having ET, but responses to treatment and results of the comprehensive DLB-consensus-based assessments, finally clarified the appropriate diagnosis (Fig. 1).

Our data thus would suggest that in some cases ET patients are actually misdiagnosed tDLB patients.

Albeit prudent considerations are needed, in the absence of neuropathology, this finding may provide additional clarity to the on-going debate [38], opposing two factions, suggesting [16–21] or denying [22–24] the possibility that ET might evolve to PD or DLB, implicitly challenging the assumption that the three do represent distinct clinical entities rather than syndromes.

While recent studies [39, 40] might further help to clarify this controversy, by adding new concepts to the debate, our concluding remark would be focused to a simple take home message: the appropriate examination and investigation of patients with tremor should not be simply addressed to motor aspects but should also consider non-motor features and specifically the core and supportive features of DLB [1–3] i.e., cognitive, visuo-spatial and dysexecutive abnormalities, RBD, and EEG abnormalities, before reaching definite conclusions.

## Electronic supplementary material

Below is the link to the electronic supplementary material.

Supplementary material 1 (DOC 36 kb)

Supplementary material 2 (DOC 35 kb)

Supplementary material 3 (JPEG 18 kb)

Supplementary material 4 (TIFF 664 kb)

## References

[1] Mc Keith IG, Dickson DW, Lowe J (2005). Diagnosis and management of dementia with Lewy bodies: third report of the DLB Consortium. Neurology.

[2] Ballard C, McKeith I, Burn D (1997). The UPDRS scale as a means of identifying extrapyramidal signs in patients suffering from dementia with Lewy bodies. Acta Neurol Scand.

[3] Aarsland D, Ballard C, McKeith I (2001). Comparison of extrapyramidal signs in Dementia with Lewy Bodies and Parkinson’s Disease. J Neuropsychiatry Clin Neurosci.

[4] Deuschl G, Bain P, Brin M (1998). Consensus statement of the movement disorder society on tremor. Ad Hoc scientific committee. Mov Disord.

[5] Deuschl G, Papengut F, Hellriegel H (2012). The phenomenology of Parkinsonian tremor. Parkinsonism Relat Disord.

[6] Jankovic J, Lang AE (2004) Movement disorders: diagnosis and assessment. In: Bradley G, Daroff RB, Fenichel GM, Jankovic J (eds) Neurology in clinical practice, 5th edn, Butterworth Heinemann Elsevier, London

[7] Roze E, Coelho-Braga MC, Gayraud D (2006). Head tremor in Parkinson’s disease. Mov Disord.

[8] Gan J, Xie-Brustolin J, Gervais-Bernard H (2009). Possible Parkinson’s disease revealed by a pure head resting tremor. J Neurol Sci.

[9] Jankovic J, Schwartz KS, Ondo W (1999). Re-emergent tremor of Parkinson’s disease. J Neurol Neurosurg Psychiatry.

[10] Leu-Semenescu S, Roze E, Vidailhet M (2007). Myoclonus or tremor in orthostatism: an under-recognized cause of unsteadiness in Parkinson’s disease. Mov Disord.

[11] Thomas A, Bonanni L, Antonini A (2007). Dopa-responsive pseudo-orthostatic tremor in parkinsonism. Mov Disord.

[12] Invernizzi F, Varanese S, Thomas A (2008). Two novel POLG1 mutations in a patient with progressive external ophthalmoplegia, levodopa-responsive pseudo-orthostatic tremor and parkinsonism. Neuromuscul Disord.

[13] Infante J, Berciano J, Sánchez-Juan P (2009). Pseudo-orthostatic and resting leg tremor in a large Spanish family with homozygous truncating parkin mutation. Mov Disord.

[14] Byrne EJ, Lennox G, Lowe J (1989). Godwin-Austen RB (1989) Diffuse Lewy body disease: clinical features in 15 cases. J Neurol Neurosurg Psychiatry..

[15] Lucetti C, Logi C, del Dotto P, Berti C, Ceravolo R, Baldacci F, Dolciotti C, Gambaccini G, Rossi G, Bonuccelli U (2010). Levodopa response in dementia with lewy bodies: a 1-year follow-up study. Parkinsonism Relat Disord.

[16] Louis ED, Faust PL, Vonsattel JP (2009). Older onset essential tremor: more rapid progression and more degenerative pathology. Mov Disord.

[17] LaRoia H, Louis ED (2011). Association between essential tremor and other neurodegenerative diseases: what is the epidemiological evidence?. Neuroepidemiology.

[18] Ceravolo R, Antonini A, Volterrani D (2008). Predictive value of nigrostriatal dysfunction in isolated tremor: a clinical and SPECT study. Mov Disord.

[19] Thawani SP, Schupf N, Louis ED (2009). Essential tremor is associated with dementia: prospective population-based study in New York. Neurology.

[20] Louis ED, Agnew A, Gillman A, Gerbin M, Viner AS (2011). Estimating annual rate of decline: prospective, longitudinal data on arm tremor severity in two groups of essential tremor cases. J Neurol Neurosurg Psychiatry.

[21] Louis ED, Asabere N, Agnew A (2011). Rest tremor in advanced essential tremor: a post-mortem study of nine cases. J Neurol Neurosurg Psychiatry.

[22] Adler CH, Shill HA, Beach TG (2011). Essential tremor and Parkinson’s disease: lack of a link. Mov Disord.

[23] Hallett M, Deuschl G (2010). Are we making progress in the understanding of tremor in Parkinson’s disease?. Ann Neurol.

[24] Quinn NP, Schneider SA, Schwingenschuh P, Bhatia KP (2011). Tremor–some controversial aspects. Mov Disord.

[25] Hughes AJ, Daniel SE, Kilford L, Lees AJ (1992). Accuracy of clinical diagnosis of idiopathic Parkinson’s disease: a clinico-pathological study of 100 cases. J Neurol Neurosurg Psychiatry.

[26] Criscuolo C, De Rosa A, Guacci A (2011). The LRRK2 R1441C mutation is more frequent than G2019S in Parkinson’s disease patients from southern Italy. Mov Disord.

[27] Silveira-Moriyama L, Lees AJ (2011). Up with the lark: a panoptic view of Parkinson disease. Neurology.

[28] Declaration of Helsinki (1997). Recommendation Guiding Physicians in Biomedical Research involving human subjects. JAMA.

[29] Bonanni L, Thomas A, Tiraboschi P, Perfetti B, Varanese S, Onofrj M (2008). EEG comparisons in early Alzheimer’s disease, dementia with Lewy bodies and Parkinson’s disease with dementia patients with a 2-year follow-up. Brain.

[30] Onofrj M, Bonanni L, Manzoli L, Thomas A (2010). Cohort study on somatoform disorders in Parkinson disease and dementia with Lewy bodies. Neurology.

[31] Onofrj M, Monaco D, Bonanni L, Onofrj V, Bifolchetti S, Manzoli L, Thomas A (2011). Eyelid retraction in dementia with Lewy bodies and Parkinson’s disease. J Neurol.

[32] Franciotti R, Falasca NW, Bonanni L et al (2012) Default network is not hypoactive in dementia with fluctuating cognition: an Alzheimer disease/dementia with Lewy bodies comparison. Neurobiol Aging. doi: 10.1016/j.neurobiolaging.2012.09.015 10.1016/j.neurobiolaging.2012.09.015 23063646

[33] Onofrj M, Thomas A, Paci C, D’Andreamatteo G (1998). Gabapentin in orthostatic tremor: results of a double-blind crossover with placebo in four patients. Neurology.

[34] Zeuner KE, Molloy FM, Shoge RO (2003). Effect of ethanol on the central oscillator in essential tremor. Mov Disord.

[35] Knudsen K, Lorenz D, Deuschl G (2011). A clinical test for the alcohol sensitivity of essential tremor. Mov Disord.

[36] Albanese A, Bonuccelli U, Brefel C (2001). Consensus statement on the role of acute dopaminergic challenge in Parkinson’s disease. Mov Disord.

[37] Lippa CF, Duda JE, Grossman M (2007). et al., DLB/PDD Working Group. DLB and PDD boundary issues: diagnosis, treatment, molecular pathology, and biomarkers. Neurology.

[38] Louis E, Adler C (2012). Is essential tremor predictive of Parkinsons disease. Mov Along.

[39] Albin RL, Dauer WT (2012). Parkinson syndrome. Heterogeneity of etiology; heterogeneity of pathogenesis?. Neurology.

[40] Helmich RC, Hallett M, Deuschl G, Toni I, Bloem BR (2012). Cerebral causes and consequences of parkinsonian resting tremor: a tale of two circuits?. Brain.

