# Supplementary material for: Cohort study of prevalence and phenomenology of tremor in dementia with Lewy bodies
Source: J Neurol. 2013 Feb 12;260(7):1731–42. doi: 10.1007/s00415-013-6853-y (PMC3705145; doi:10.1007/s00415-013-6853-y)
Supplement: Supplementary file 1 — Supplementary material 1 (DOC 36 kb) [file 415_2013_6853_MOESM1_ESM.doc]

**Online Resource 1.** *Clinical, electrophysiological and pharmacological evaluations at enrollment.*

All patients enrolled in the study were interviewed regarding their medical and pharmacological history, and received a full neurological examination. Presence and severity of extrapyramidal signs were rated using the Unified Parkinson’s Disease Rating Scale (UPDRS)[1] and the Hoehn and Yahr scale (H/Y)[2]. Patients with concomitant dystonia were excluded from the study as were patients already affected by Parkinson’s Disease with Dementia (PDD) at the baseline assessment. In addition all patients previously exposed to neuroleptics, or alchool/substances abuse, were also excluded from the study.

*Neuroimaging*

Each patient underwent a Computerized Tomography (CT) scan and Magnetic Resonance Imaging (MRI) and dopaminergic presynaptic ligand ioflupane Single Photon Emission Computer Tomography (SPECT) Dopamine Transporter. To be enrolled in the study SPECT scans had to be abnormal in DLB and PD patients and normal in ET patients.

*Neurogenetics*

After obtaining written informed consent, patients underwent blood sampling and DNA extracted following standard procedures. Exons 41, 29, 31 and 48 of the LRRK2 gene, including exon-intron junctions were bidirectionally sequenced using the Big Dye Terminator chemistry and an ABI PRISM 3130XL automated sequencer (Applied Biosystem; primers and conditions available upon request). No pathogenic mutations or rare variants were identified in the patients’ cohort.

*Neuropsychological evaluation*

Global test of cognition included Mini Mental State Examination (MMSE)[3] and Dementia Rating Scale-2 (DRS-2)[4]. Frontal and dysexecutive symptoms were evaluated with the Frontal Assessment Battery (FAB).[5]

*Neuropsychiatric evaluation*

The presence of visual hallucinations (VH) or other psychotic symptoms was assessed by the Neuropsychiatric Inventory[6]. Presence and severity of cognitive fluctuations were evaluated using Clinician Assessment of Fluctuations (CAF) scores.[7] In addition suspected DLB patients underwent quantitative electroencephalographic (EEG). We have previously demonstrated that the presence of variability of dominant frequencies or presence of dominant frequencies at 6.0-7.9 Hz in the absence of a clear posterior alpha frequency are features associated with DLB and thus EEG analysis was used to support the diagnosis.[8]

The presence of REM Sleep Behaviour Disorder (RBD) was evaluated according to minimal International Classification of Sleep Disorders (ICD-10) criteria for RBD[9] and confirmed by polysomnographic recordings.

*EMG recording of tremor*

Bipolar 9 mm Ag/AgCl needle electrodes placed 2 to 4 cm apart over the muscle bellies as described in previous studies. Simultaneous recordings were obtained from both tibiales anteriores, gastrocnemii mediales, vasti mediales, bicipites femoris, extensors and flexors carpi ulnares. The recordings included scaleni and cervical paraspinals for head tremor, and mentalis muscle for face (chin) tremor (‘face tremor’ is used in this paper to indicate chin tremor, according to TRGRS scale indications). Band pass filters were set at 0.5 to 5,000 Hz and gain was 20,000 Hz; the acquired signals were processed with Galileo Units (Eb Neuro Florence) performing fast Fourier transform frequency analysis (FA) of 32 repeated 2-second epochs with 1024 Hz sampling rate. Dominant frequencies were represented as peaks of amplitudes in using a Compressed Spectral Array method ^8^. The patients were recorded while sitting in relaxed position, with arms comfortably resting on their lap. In addition patients were asked to sit with arms extended in forward horizontal reach posture and also while standing.

**e-References**

1. Fahn S, Elton RL. Members of the Unified Parkinson’s Disease Rating Scale Development Committee. Unified Parkinson’s disease rating scale. In: Fahn S, Marsden CD, Calne DB, Goldstein M, editors. Recent development in Parkinson’s disease, Vol. 2 Florham Park, New Jersey: Macmillan Healthcare Information: 1987, pp. 153-164.
2. Hoehn MM, Yahr MD. Parkinsonism: onset, progression and mortality. Neurology 1967;17:427-442.
3. Folstein NF, Folstein SE, McHugh PR. Mini-mental state: a practical method for grading the cognitive state of patients for clinician. J Psychiatry Res 1975;12:189-198.
4. Jurica PJ, Leitten CL, Mattis S. DRS-2 Dementia Rating Scale 2. Psychological Assessment Resources Eds. 2001.
5. Dubois B, Slachevsky A, Litvan I, Pillon B. The FAB: a Frontal Assessment Battery at bedside. Neurology 2000;55:1621-1626.
6. Cummings JL, Mega M, Gray K, Rosenberg-Thompson S, Carusi DA, Gornbein J. The Neuropsychiatric Inventory: comprehensive assessment of psychopathology in dementia. Neurology 1994;44:2308-2314.
7. Walker MP, Ayre GA, Cummings JL, et al. The Clinician Assessment of Fluctuation and the One Day Fluctuation Assessment Scale. Two methods to assess fluctuating confusion in dementia. Br J Psychiatry 2000;177:252-256.
8. Bonanni L, Thomas A, Tiraboschi P, Perfetti, B, Varanese S, Onofrj M. EEG comparisons in early Alzheimer's disease, dementia with Lewy bodies and Parkinson's disease with dementia patients with a 2-year follow-up. Brain 2008;131:690-705.
9. World Heath Organization. The ICD-10 classification of mental and behavioural disorders. Geneva: WHO, 1992
