# Supplementary material for: Cohort study of prevalence and phenomenology of tremor in dementia with Lewy bodies
Source: J Neurol. 2013 Feb 12;260(7):1731–42. doi: 10.1007/s00415-013-6853-y (PMC3705145; doi:10.1007/s00415-013-6853-y)
Supplement: Supplementary file 2 — Supplementary material 2 (DOC 35 kb) [file 415_2013_6853_MOESM2_ESM.doc]

**Online Resource 2.** *Additional information on tremors observed in the groups.*

Head tremor, with severity score at the TRGS of 1-2, was observed in 3.1% of tPD, 26.7% of tDLB, and 29.4% of ET patients. It consisted of the classical “no-no” rotational pattern in PD and DLB. This pattern was also observed in 23% of ET patients although a “yes-yes” pattern was observed in 11.7% ET patients. In 2 PD and 6 DLB patients intentional head rotation abolished tremor, which restarted after reaching the end of rotation^1-3^. In 10 ET patients head tremor persisted during head rotation. The frequency of bursts in paraspinal and scaleni muscles was in the 2.3-4.5 Hz range independently of DLB, PD or ET diagnosis.

Voice tremor with bleating voice, unchanged by gestures or singing, was observed only in 6 ET patients (17.6%) with severity score at the TRGS ranging from 1 to 3.

Face tremor at 5.2-7.1 Hz, involving mentalis, triangularis and quadratus labii muscles was observed in 1.8% of PD, 16.4% of DLB, 14.7% of ET patients.

Head and chin tremor in PD and DLB was always concomitant with rest and postural tremors of arms: 6 DLB patients with head tremor and 4 DLB patients with face tremor had the mixed tremor pattern described below. Head, chin and voice tremor in ET patients was always concomitant with arm tremor (because of subject selection criteria), but never with jaw tremor, rhythmic clicking of teeth, perioral and nasal muscle.

Tremulous DLB and PD patients affected by the standing tremor found the the standing position intolerable for any period of time, but the leg tremor disappeared when walking, similarly to orthostatic tremor. In 11 of the 16 tDLB patients presenting with standing legs tremor, walking abolished the leg tremor but elicited arms tremor with identical frequency as the rest tremor. During outstretched arms standing posture, the tremor in arms or face did not increase, but tremor in lower limbs increased from a score of 0 to 3 on the TRGRS. The amplitude of tremor in the different body districts was increased by the standing posture in 6 tDLB patients and the two tPD patients: the amplitude incremented from a score of 1 to 3 when comparing rest with postural tremor in stretched arms, and from 0 to 3 in lower limbs.

During standing with arms stretched in a wing-beating posture an isolated re-emergent tremor, with same amplitude and frequency as the concomitant rest tremor, was observed in 13.5% of tPD and 4.5% of tDLB patients. This tremor was considered as the specific re-emergent parkinsonian tremor^4^ as it was not accompanied by intentional tremor, and it had the same frequency as rest tremor (5.3-7.1 Hz rest, 5.6- 7.2HZ re-emergent). The latency for re-emergency of the rest tremor in the 15 PD and 3 DLB patients was 3.6±1.0 sec, with a range 2 to 12 sec.

**e-References**

1. Roze E, Coelho-Braga MC, Gayraud D et al. Head tremor in Parkinson's disease, Mov Disord 2006; 21:1245–1248.
2. Gan J, Xie-Brustolin J, Gervais-Bernard H, Vallet AE, Broussolle E, Thobois S. Possible Parkinson's disease revealed by a pure head resting tremor. J Neurol Sci 2009; 279:121-123.
3. Caviness JN, Adler CH, Beach TG, Wetjen KL, Caselli RJ. Small-amplitude cortical myoclonus in Parkinson's disease: physiology and clinical observations. Mov Disord. 2002;17:657-662.
4. Jankovic J, Schwartz KS, Ondo W. Re-emergent tremor of Parkinson’s disease. J Neurol Neurosurg Psychiatry 1999;67:646-650.
