# Supplementary figures and images for: Cohort study of prevalence and phenomenology of tremor in dementia with Lewy bodies
Source: J Neurol. 2013 Feb 12;260(7):1731–42. doi: 10.1007/s00415-013-6853-y (PMC3705145; doi:10.1007/s00415-013-6853-y)

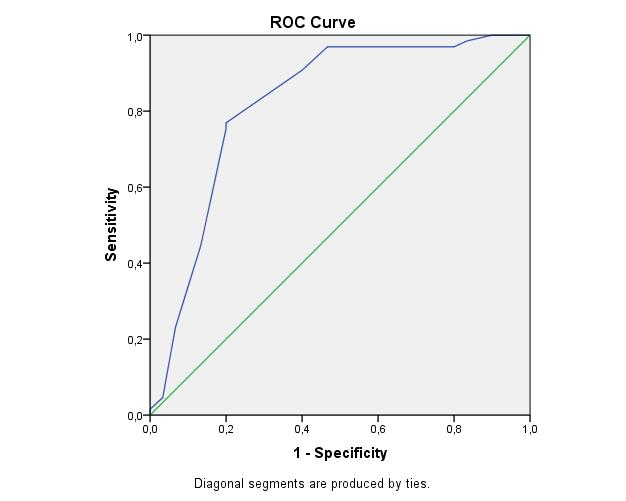

Supplement: Supplementary file 3 — Supplementary material 3 (JPEG 18 kb) [file 415_2013_6853_MOESM3_ESM.jpg]

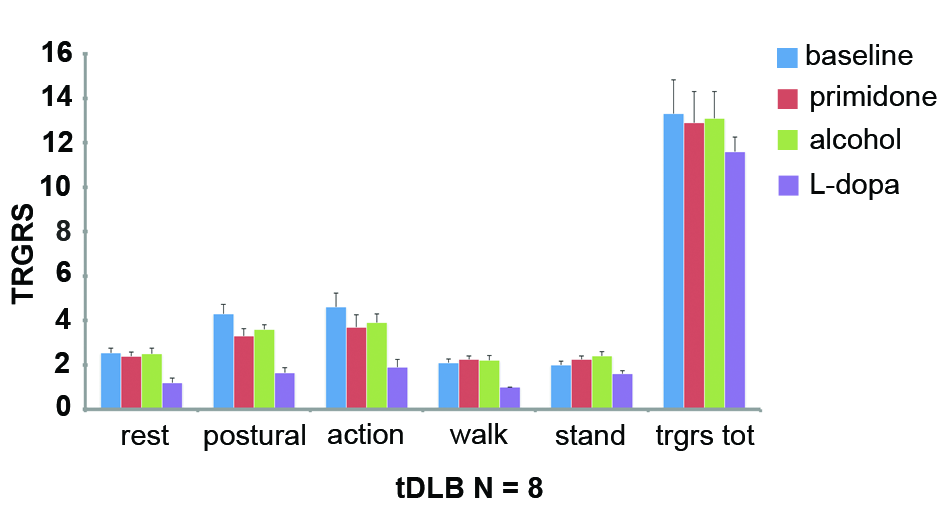

Supplement: Supplementary file 4 — Supplementary material 4 (TIFF 664 kb) [file 415_2013_6853_MOESM4_ESM.tif]
